# Neuroendocrine tumours and pregnancy: Real‐world data from an European Neuroendocrine Tumour *Centre of Excellence*

**DOI:** 10.1111/jne.13465

**Published:** 2024-11-06

**Authors:** Gowri M. Ratnayake, Kalyan Mansukhbhai Shekhda, Thomas Glover, Yasser Al‐Obudi, Aimee Hayes, Panagiotis Armonis, Dalvinder Mandair, Bernard Khoo, TuVinh Luong, Christos Toumpanakis, Ashley Grossman, Martyn Caplin

**Affiliations:** ^1^ Neuroendocrine Tumour Unit, ENETS Centre of Excellence Royal Free Hospital London UK; ^2^ Department of Endocrinology and Diabetes University College London Hospitals London UK; ^3^ Department of Radiology Royal Free Hospital London UK; ^4^ Department of Pathology Royal Free Hospital London UK

**Keywords:** neuroendocrine tumours, oestrogen, pregnancy, somatostatin analogues

## Abstract

Neuroendocrine neoplasms (NENs) arise from the diffuse endocrine system and have been considered to be rare. However, the incidence and prevalence of these tumours have increased in recent years, and they are being seen in younger patients including women in the reproductive age group. Due to the paucity of data, diagnostic and therapeutic strategies in managing such tumours during pregnancy can be challenging to both treating physicians and patients. This article describes the experience and outcomes of managing pregnant women with NEN at a *European Neuroendocrine Tumour Society (ENETS) Centre of Excellence*. In this retrospective analysis, we evaluated a total of 22 pregnancies in 18 pregnant women with concurrent diagnoses of NENs who were managed at Royal Free Hospital *ENETS Centre of Excellence* throughout their pregnancy. These were identified from our tumour registry of 3500 NEN patients between 2015 and 2023. Cross‐sectional imaging (computed tomography (CT)/magnetic resonance imaging (MRI)), pre‐ and post‐pregnancy, for each patient was reviewed by an experienced radiologist. Tumour growth rate (TGR) was calculated using the formula: TGR = 100 × [exp (TG) − 1]; TG. [3 × log (D2/D1)]/time (months), where D1 is the tumour size at date 1; D2 is the tumour size at date 2; and time (months) = (Date 2 − Date 1 + 1)/30.44. Tumour growth rate pre‐conception (TGRpc) and tumour growth rate post‐partum (TGRpp) were calculated for each patient. In a sub‐group of patients, positivity for oestrogen and progesterone receptors were analysed on the tumour tissue to evaluate whether the presence of these receptors affected tumour progression during the pregnancy. We also reviewed the pregnancy outcome in patients treated with somatostatin analogues during pregnancy. We analysed the data of a total 22 pregnancy encounters in 18 women: 15 pregnancies (68%) preceded the diagnosis of the NEN, whereas the diagnosis of NEN was made during pregnancy or in the post‐partum period in 5 (23%) and 2 (9%) pregnancies respectively. Eight patients (44%) had a diagnosis of a pancreatic NEN, whereas 5 (28%) were diagnosed with mid‐gut NENs, and a further 5 at other sites. The majority of the patients (*n* = 12, 67%) had evidence of metastatic disease at the time of diagnosis. Most pregnancies had a successful outcome (*n* = 19, 86%), whereas 3 patients (14%) had miscarriages in the 1st trimester. Five patients in total of 6 pregnancies were treated with somatostatin analogues as monotherapy during the pregnancy, and all of them had stable disease after pregnancy. All of them delivered healthy babies without any side effects or complications due to therapy. The average TGRpc was −0.8% (*n* = 5) and the average TGRpp was +0.96% (*n* = 6); 2 patients who did not have suitable targets for calculation of TGRpc developed new lesions suggesting disease progression. Moreover, 2 of the 4 patients who have had both pre‐conception and post‐pregnancy scans showed an increase in TGRpp compared to TGRpc. The management of NENs during pregnancy should be multidisciplinary with an individualised approach to each patient. Somatostatin analogues appear to be safe during pregnancy, though further robust studies are needed. Pregnancy per se may accelerate tumour progression, and patients should be counselled regarding this possibility.

## 1 INTRODUCTION

Neuroendocrine neoplasms (NENs) comprise a heterogeneous group of tumours arising from the diffuse endocrine system.^1^ Thus, NENs are reported at various locations and may be sub‐categorised as gastro‐enteropancreatic (GEP), bronchial, thymic, pituitary, renal and ovarian NENs, although other sites have been reported. Furthermore, they may be either functional, secreting one or more hormones associated with a syndrome, or non‐functional with no associated hormonal syndrome.^2^ NENs may also present as a part of a familial genetic syndrome, usually autosomal dominant, such as multiple endocrine neoplasia types 1 and 4 (MEN1 and MEN4), Von‐ Hippel‐Lindau (VHL) syndrome, von Recklinghausen disease (neurofibromatosis type 1 or NF‐1) and tuberous sclerosis.

Although the diagnosis of NENs in younger populations (<50 years) is less common, its incidence has increased over the years.^3^ The age‐standardised rate (ASR) of incidence of NENs among patients <54 years old who were diagnosed between 2013 and 2015 was 1.88 per 100,000 population in the UK.^4^ Moreover, a retrospective study analysing the incidence survival of NENs in England revealed that the incidence of NENs has risen 3.7‐fold in England over the period between 1995 and 2008, while the median age of appendiceal NENs was only 39 years (IQR: 25–58).^5^ Another study, based on the National Cancer Database in the period of 2004–2016 in the USA, reported that young‐onset (age <50 years) pancreatic NENs accounted for 27% of all patients with pancreatic NENs, the median age in this age group being 42 years.^6^ Thus, females in the reproductive age range are increasingly being diagnosed with NENs. Furthermore, the maternal age at pregnancy has increased in recent decades, and therefore the number of women becoming pregnant while diagnosed with cancer in general has increased.^7^ Additionally, it is estimated that 1 in every 1000 pregnant women is first diagnosed with cancer during their pregnancy.^8^

The symptomatology of NENs varies according to their anatomical location and to the functionality of the tumour, including the secretion of vasoactive factors such as serotonin and bradykinin into the systemic circulation. The ‘gold standard’ for the diagnosis of NENs is histopathological examination of the tumour tissue, and the Ki‐67 index of the specimen is used for tumour grading.^9^ Circulating markers such as chromogranin A levels (albeit with a sensitivity of only 71.3%–83% and specificity of 71%–85%)^10^ and more recent transcriptomic biomarkers such as the NETest (sensitivity 94.4% and specificity of 95.4%, but still not easily available), may also be used to aid in the diagnosis of NENs.^1^ Cross‐sectional imaging modalities such as contrast‐enhanced CT (overall sensitivity 82% and specificity 86%) and MRI (sensitivity 70%–79% and specificity 98%–100%) are used for diagnosis, monitoring the response to treatment for NENs, and for their surveillance.^11^ Functional somatostatin receptor (SSTR) imaging with, for example, ^68^Gallium‐DOTATATE‐PET/CT scanning, can be used in the diagnosis and staging of SSTR‐positive NENs,^1^, ^11^ and additionally has theranostic value to determine appropriate patients for peptide‐receptor radionuclide therapy (PRRT).

The treatment of advanced well‐differentiated SSTR‐avid NENs includes long‐acting SSTR analogues, PRRT and liver‐targeted treatments, for example, embolisation and ablation, whilst other therapies include biological targeted therapies (everolimus and sunitinib), occasionally interferon‐α^12^, ^13^, ^14^ and chemotherapy.^14^ More recently, there is limited evidence that immune checkpoint inhibitor therapy is also effective in some patients with high‐grade NENs, although these agents are not yet recommended in standard clinical practice guidelines.^1^, ^14^

The evidence base for these diagnostic and therapeutic strategies in pregnant women is limited, since pregnant women are excluded from most clinical trials,^8^ and there is caution regarding unnecessary exposure of the mother and foetus to ionising radiation, and the rarity of NENs in this population renders experience in most centres to be limited.^15^ The management of NENs during pregnancy and women with requirements for fertility is challenging, both in terms of the safety of various therapies in pregnant women, as well as their efficacy, and their effects on reproductive capacity. In addition, the effect of pregnancy on the stability or progression of NENs is unclear. As a *Centre of Excellence* specialising in NETs, we have retrospectively assessed the impact of diagnostic and therapeutic manoeuvres in pregnant women with a concurrent diagnosis of a NEN, and discuss the available literature on follow‐up and treatment of these women during pregnancy and their fertility concerns.

## 2 METHODS

A retrospective analysis was carried out in all the pregnant patients managed at the Royal Free Hospital ENETS *Centre of Excellence*, identified from our central database. These were identified from our tumour registry of over 1800 NEN patients under active surveillance from our database of over 3500 NEN patients between 2015 and 2023. This includes patients who were diagnosed with an NEN prior to pregnancy, during pregnancy, and within 6 months of delivery. The patients were monitored during the pregnancy with ultrasound scans and, when necessary, with non‐contrast MRI scans, at frequencies decided by a multi‐disciplinary team. All patients who were diagnosed with a NEN before the pregnancy had cross‐sectional imaging for tumour surveillance at least within 4 months of conception, and again within 6 months of delivery.

Cross‐sectional imaging (CT/MRI) performed prior to conception and following delivery for each patient was reviewed by one radiologist with experience in NETs, blinded to the clinical data.

Tumour growth rate (TGR) was calculated using an adapted version of RECIST 1.1,^16^, ^17^ which allowed inclusion of a maximum of 10 target lesions (5 per organ) and used RECIST 1.1 criteria to define and measure lesions. The calculation of TGR was expressed as the percentage change in tumour volume over 1 month (%/m) and was calculated using the formula: TGR = 100 × [exp (TG) − 1]; TG. [3 × log (D2/D1)]/time (months), where D1 is the tumour size at date 1; D2 is the tumour size at Date 2; and time (months) = (Date 2 − Date 1 + 1)/30.44; TG is the tumour growth.^16^, ^17^, ^18^, ^19^ The sum of the longest diameters (SLD) of the target lesions was used to calculate tumour size (D1, D2).

Tumour growth rate *pre‐conception* (TGRpc) and tumour growth rate post‐partum (TGRpp) were calculated for each patient, where possible. TGRpc was calculated by comparing the latest scan performed prior to conception (C − 1) with the scan immediately preceding it (C − 2); TGRpp was calculated by comparing the first post‐partum scan (P + 1) with the subsequently performed scan (P + 2). Statistical analysis for correlation between pre‐pregnancy interval scan and TGRpc was performed using Pearson correlation coefficient in SPSS (version 23, IBM) and the *p* value of <.05 was considered to be statistically significant.

As this study was retrospective audit of practice, ethical approval was not required under the UK Policy Framework for Health and Social Care Practice.

## 3 RESULTS

Table 1 summarises the data related to the neuroendocrine tumour and maternal and foetal outcomes of the patients. There were 18 pregnant patients who had a concurrent diagnosis of a well‐differentiated NEN, while 4 of these patients in the cohort were pregnant twice. Therefore, a total of 22 pregnancies with a concurrent NEN diagnosis were included in this analysis. The mean age at the diagnosis of the NEN (*N* = 18) was 29.5 years (SD = 7) and their mean age during the pregnancies (*N* = 22) was 33.5 years (SD = 4.2). A total of 15 pregnancies (68%) preceded the diagnosis of the NEN, while a diagnosis of NEN was made during pregnancy or in the post‐partum period in 5 (23%) and 2 (9%) pregnancies respectively.

**TABLE 1:** Disease characteristics and the pregnancy outcomes of the cohort.

Patient no.	Age at NEN diagnosis (Years)	Site of primary tumour	Sites of disease during pregnancy	Ki 67	Grade	Age at Pregnancy (Years)	Treatments before pregnancy	Treatments during pregnancy	Antenatal period	Pregnancy outcome	Mode of delivery	POA at delivery (Weeks)	Birth weight (kg)	Postnatal /postpartum complications	NEN status post delivery
1	35	Mid gut (carcinoid syndrome)	Liver, mesenteric LN, bone	<1%	G1	39	Surgery	None	None	Successful	NVD	38	N/A	None	Progressive
2B	33	Pancreas (Gastrinoma)	Liver, pancreas	5%	G2	36	PPIs	PPIs	None	Successful	CS (breech)‐ Elective	38	3.4	None	Progressive
3	33	Mid gut (carcinoid syndrome) ER/PR negative	Liver and pancreas	3%	G2	35	Surgery, Octreotide LAR	Octreotide LAR	PPROM	Successful	CS‐ Emergency	31	2.13	IV at NICU, NG tube feeding, ROP, Ventriculomegaly Mild developmental delay in the baby	Stable
4	26	Pancreatic ER +ve 40%	Liver, abdominal node, pancreas	Variable up to 20%	G2	31	Octreotide LAR	Octreotide LAR	None	Successful	CS‐ Elective	38	4.3	None	Progressive
5	34	Bronchial (carcinoid syndrome)	Nil	3%	G2	38	Surgery	None	None	Miscarriage	N/R	10	N/R	None	Stable
6A	31	Mid gut ER +ve 30%, PR −ve	Liver	<2%	G1	34	Surgery^a^ and RFA	None	None	Successful	NVD	40	3.9	None	Progressive
7	16	Carotid body paraganglioma (Bilateral) Focal +ve ER/PR (<10%)	Solitary residual carotid body tumour in left neck	N/A	N/A	39	Surgery, Chemotherapy	None	Hypotension with epidural	Successful	NVD	39	4.08	None	Stable
8	14	Renal	Renal bed, lung, anterior abdominal wall	35%	G3	25	Surgery	None	None	Successful	NVD	38	N/A	None	Progressive
9 A^b^	37	Pancreas	Pancreas	N/A	N/A	36	Cabergoline	None	None	Miscarriage	N/R	8	N/R	None	Stable
9 B	37	Pancreas	Pancreas	N/A	N/A	37	None	None	None	Successful	NVD	38	3.52	DVT in the patient	N/A
10 A	21	Mid gut (carcinoid syndrome)	Liver, mid gut, portocaval, pelvic and retroperitoneal LN	5%	G2	33	Octreotide LAR	Octreotide LAR (not in 1st trimester)	Anaemia, GDM	Successful	CS	38	2.56	None	Stable
10 B	21	Mid gut (carcinoid syndrome)	Liver, mid gut, portocaval, pelvic and retroperitoneal LN	5%	G2	36	Octreotide LAR	Octreotide LAR	None	Successful	CS	38	2.5	None	N/A
11	27	Bronchial (carcinoid syndrome)	Lymphnodes (subcarinal lymphnodes)	<2%	G1	28	N/R	Ocreotide LAR (Not in First trimester)	None	Successful	CS	38	3.7	None	Stable disease
12	33	Bronchial (carcinoid syndrome)	Nil	4.4%	G2	33	N/R	N/R	None	Successful	Forceps delivery	38	3.56	None	No metastatic disease seen
13	31	Metastatic Well differentiated Pancreatic NET (Ki 67: 5%)	Liver	5%	G2	33	Lanreotide LAR, PRRT within a year before pregnancy	Lanreotide LAR	Gestational Diabetes Diet controlled	Successful	CS	39	3.3	None	Partial response: which was also noted after PRRT therapy before pregnancy
14	28	Pancreas	Pancreas	1%–2%	G1	28	N/R	None	None Foetal distress	Successful	CS ‐Emergency	32	1.64	NIV at NICU	Stable
15	33	Mid gut	Liver, mesenteric LN, mid gut	4%–5%	G2	33	N/R	None	None	Successful	CS‐ Elective	34	2.44	IV at NICU	Stable
16	40	Pancreas	Pancreas	<1%	G1	40	N/R	None	None	Successful	NVD	38	N/A	None	Stable
17	35	Pancreas (Insulinoma)	Bone, pancreas	<3%	G1	35	N/R	SC Octreotide	Severe hypoglycaemia	Successful	NVD	40	3.18	None	Progressive
2 A	33	Pancreas (gastrinoma)	Liver, pancreas	5%	G2	33	N/R	PPIs	None	Miscarriage	N/R	25	N/R	None	Progressive
18^c^	25	Pancreas	Liver, pancreas	12%	G2	25	N/R	N/R	Preterm Labour	Successful	CS‐ Emergency	29	1.42	Cerebral palsy	N/R
6B	31	Mid gut	Liver, mid gut	<1%	G1	31	N/R	N/R	None	Successful	NVD	40	3.7	None	N/R

*Note*: A: indicates first pregnancy. B: indicates second pregnancy in the same patient. 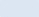 Patients who had NEN diagnosed before pregnancy. 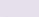 Patients who were diagnosed with NEN during pregnancy. 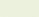 Patients who were diagnosed with NEN in the postpartum period.

Abbreviations: CS, Caesarean section; GDM, gestational diabetes mellitus; IV, invasive ventilation; NA, not available; NICU, neonatal intensive care unit; NIV, non invasive ventilation; NR, not relevant; NVD, normal vaginal delivery; PPIs, proton pump inhibitors; PPROM, preterm pre labour rupture of membranes; ROP, retinopathy of prematurity.

^a^ Surgical resection of the primary tumour, multiple wedge resection and right hepatectomy.

^b^ Has MEN 1 syndrome and was treated with 3 gland parathryoidectomy before the 1st pregnancy.

^c^ Simultaneous diagnoses of breast cancer and PTHrP related hypercalcaemia.

There were 28% (*n* = 5) of the patient group with midgut NENs, 44% (*n* = 8) with pancreatic NENs and 5 patients with either bronchial, paraganglioma or renal NENs. The majority, 67% (*n* = 12), had evidence of metastatic disease at the time of diagnosis. Of 5 patients with mid‐gut NENs, two patients had grade 1 NENs with Ki67 of <2%, three patients had grade 2 NENs (Ki67 of 3%–5%). Of the 8 patients with pancreatic NENs, 4 patients had grade 2 NENs with Ki67 5%–20%, 3 patients had Grade 1 tumours, while the histological NEN grading was not available for the remaining patient. Of those with bronchial NENs (*n* = 3), 2 had grade 2 NENs with Ki 67 of 3% and 4.4% and another had a grade 1 NEN. One patient had a metastatic paraganglioma but histological tumour grading was not available. The patient with a renal NEN had a G3 tumour with a Ki 67 of 35%. Eight patients had functional NENs with 6 having carcinoid syndrome, one had a gastrinoma while another had an insulinoma. Most of the patients were treated with resection of the primary tumour and regional metastatic disease. Patient no. 6 underwent resection and radiofrequency ablation of liver metastases before conception. None of the patients was on chemotherapy at the time of conception or had received it a year before conception. Three patients (patient nos. 3, 4 and 10) were on octreotide LAR therapy every 28 days at conception. Patient no. 10 stopped the octreotide LAR injection only during the 1st trimester in her first pregnancy but she opted to continue Octreotide LAR throughout the whole of her second pregnancy, while patient nos. 3 and 4 continued octreotide LAR throughout their pregnancies as these patients also had a carcinoid syndrome. Although patient no. 11 was not on Octreotide LAR therapy at the time of conception, she was started on Octreotide LAR every 28 days in the 2nd trimester. Fourteen of the 15 (93%) had residual disease at the time of conception.

Patient no. 7 was diagnosed with bilateral carotid body paragangliomas at the age of 16 years and was found to have a variant of unknown significance in the *SDH‐D* gene. Her plasma metanephrines were normal. She underwent resection of her paragangliomas on multiple occasions and chemotherapy prior to pregnancy, and only had a small volume of residual disease at pregnancy. She did not require any medications during pregnancy, although she became hypotensive after epidural anaesthesia in the peripartum period. She delivered a healthy‐term baby weighing 4.08 kg.

Most patients had an uneventful antenatal course. However, patient no. 17, with severe hypoglycaemic episodes during the first trimester due to an underlying insulinoma, was treated with subcutaneous short‐acting octreotide injections 100–200 μg subcutaneously twice‐daily, and underwent distal pancreatectomy and splenectomy at 9 weeks of gestation in a centre outside the UK. None of the patients with a carcinoid syndrome demonstrated any aggravation of their symptoms. Patient no. 10 had anaemia requiring blood transfusion and gestational diabetes during her first pregnancy, whereas patient no. 3 had preterm pre‐labour rupture of membranes at 31 weeks. Patient no. 13 was diagnosed with gestational diabetes controlled with diet alone. Patient no. 13 had completed four cycles of PRRT with ^177^Lutetium‐DOTATATE 10 months preceding pregnancy for progressive metastatic pancreatic NEN. She had a partial response to PRRT with reduction in the size of her pancreatic NET from 42 mm to 24 mm.

The majority of pregnancies, 19 (86%) had a successful outcome, although 3 (14%) had miscarriages in the 1st trimester. Of the successful pregnancies, 4 of 19 (21%) had preterm deliveries (baby born alive before 37 weeks of gestation), in 3 of them due to obstetric reasons (Table 1). The mean gestational age at the time of delivery was 37 weeks (SD = 3.1). The mean birthweight was 3.08 kg (SD = 0.86). The rate of Caesarean section was 45%, of which 6 patients of a total 7 pregnancies (1 patient twice) underwent elective Caesarean sections. Patient no. 12 underwent forceps delivery.

Three neonates required to be cared for in a neonatal intensive care unit due to issues related to prematurity; the baby of patient no. 3 had ventriculomegaly, retinopathy of prematurity, recurrent ‘glue ear’ and hearing impairment, and currently suffers from mild global developmental delay. Post‐pregnancy staging scans revealed 7 of the 17 pregnancies (41%) were associated with progressive disease, whereas patient no.13 had a partial response having received PRRT within a year preceding her pregnancy.

In this study group, SSTA (somatostatin analogues) such as Octreotide‐LAR (Octreotide long‐acting release) and Lanreotide‐LAR (Lanreotide long‐acting release) were continued in 4 patients (patients nos. 3, 4, 10 and 13) during their pregnancy. Patient nos. 3, 4 (Octreotide‐LAR), and 13 (Lanreotide‐LAR) continued it throughout the pregnancy, while patient no. 10 was treated with Octreotide‐LAR from second trimester of her first pregnancy and throughout her second pregnancy. The key indication for continuation of SSTA in patient nos. 3, 10 and 13 was the presence of the carcinoid syndrome. Although patient nos. 4 and 13 delivered healthy babies with a birthweight of 4.3 kg and 3.3 kg, respectively, no. 3 had a preterm delivery at 33 weeks due to premature rupture of membranes, and as noted above the baby had complications largely related to prematurity and ventriculomegaly. Moreover, 3 of the 4 patients who received SSTA during pregnancy had stable disease. Patient no. 11 was not on SSTA before the pregnancy but was initiated on Octreotide LAR therapy during the second trimester due to her carcinoid syndrome, and to prevent progression of the disease during pregnancy. She delivered a healthy baby weighing 3.7 kg and had stable disease during and after delivery. Patient no. 13 was on Lanreotide LAR before pregnancy which was continued throughout her pregnancy. She delivered a healthy baby of 3.3 kg weight at 39 weeks of pregnancy. She exhibited a partial response to the therapy.

Of the 5 patients diagnosed with NENs *during* pregnancy, all were asymptomatic but had detectable abnormalities on obstetric ultrasound (e.g., liver metastases) which ultimately led to the diagnosis of a NEN, except patient no. 2 with a gastrinoma who was admitted with a duodenal perforation at 16 weeks of pregnancy requiring emergency laparotomy. Patient no. 18 was detected to have liver metastases during an emergency caesarean section performed at 29 weeks for preterm labour and foetal distress, which lead to a concurrent diagnosis of a pancreatic NEN, breast cancer, and parathyroid hormone‐related peptide‐induced hypercalcaemia. On the other hand, patient no. 6B was detected with a mid‐gut NEN 4 months after the delivery due to persistent indigestion symptoms, and a loss of appetite which had appeared during early pregnancy.

The TGRs are illustrated in Table 2. Only 7/13 patients who had NENs diagnosed before becoming pregnant were included in the TGR analysis, either due to scan images being unavailable or the absence of target lesions as defined by RECIST 1.1 criteria. One patient who had PRRT within a year preceding the pregnancy was also excluded from the TGR calculation. Only 4 of the included patients had both a TGRpc and TGRpp assessment: 2 patients (patient nos. 1 and 6) did not have suitable target lesions on pre‐pregnancy scans but developed new lesions on post‐partum scans allowing the calculation of TGRpp. The remaining patient (Patient 11) had pre‐pregnancy scans allowing the calculation of TGRpc but did not have scans post‐delivery. In addition, patient nos. 1, 4 and 6 were noted to have a new metastatic lesion on their post‐pregnancy scan suggestive of a progressive NEN during the pregnancy.

**TABLE 2:** Pre and post‐pregnancy TGR.

Patient no.	Interval between pre‐pregnancy scans (months)	Pre‐pregnancy TGR (%)	Interval between post‐pregnancy scans (months)	Post‐pregnancy TGR (%)
1	‐	No suitable targets	11.27	0
3	7.65	1.50	9.56	2.91
4	21.19	1.10	7.69	0.33
6	‐	No suitable targets	11.10	1.02
8	10.64	−7.79	15.31	1.52
10B	14.98	1.19	No data	No data
11	1.91	0	2.33	0

In pre‐pregnancy scans, of 7 patients, 3 patients (patient nos. 3, 4 and 10B) had a positive TGR, one patient (patient no. 11) had 0 TGR, and one patient (patient no. 8) had negative TGR. Two patients (patient nos. 1 and 6) did not have suitable target lesions for TGR calculation. However, there was no statistical correlation between the interval from the pre‐pregnancy scan and the TGR. In post‐pregnancy scans, of these 7 patients, four patients (patient nos. 3, 4, 6 and 8) had a positive TGR, 2 patients (patient nos. 1 and 11) had 0% TGR. One patient (patient no. 10B) did not have scan to calculate TGR. The TGR had increased in 4 patients of 7 patients, and the average TGR pre‐pregnancy was −0.8% (*n* = 5) and the average post‐pregnancy TGR was 0.96% (*n* = 6). Four of the 7 patients (patient nos. 3, 4, 8 and 11) had both TGRpc and TGRpp calculated: For this cohort, the average TGRpc was −1.3% (*n* = 4), and the average TGRpp was 1.19% (*n* = 4).

In several cases, new lesions arose, or pre‐existing lesions became measurable as target lesions, during the pregnancy period, precluding the calculation of an approximate intra‐pregnancy TGR. For two of the patients (patient nos. 1 and 8), the TGR increased from pre‐ to post‐partum; notably patient no. 8 had a positive TGR post‐partum having had a negative rate pre‐pregnancy. Hence, post‐pregnancy there was a rise in the TGR with patients showing a shift from stable or regressing disease pre‐conceptually to progressing post‐pregnancy.

**FIGURE 1:**
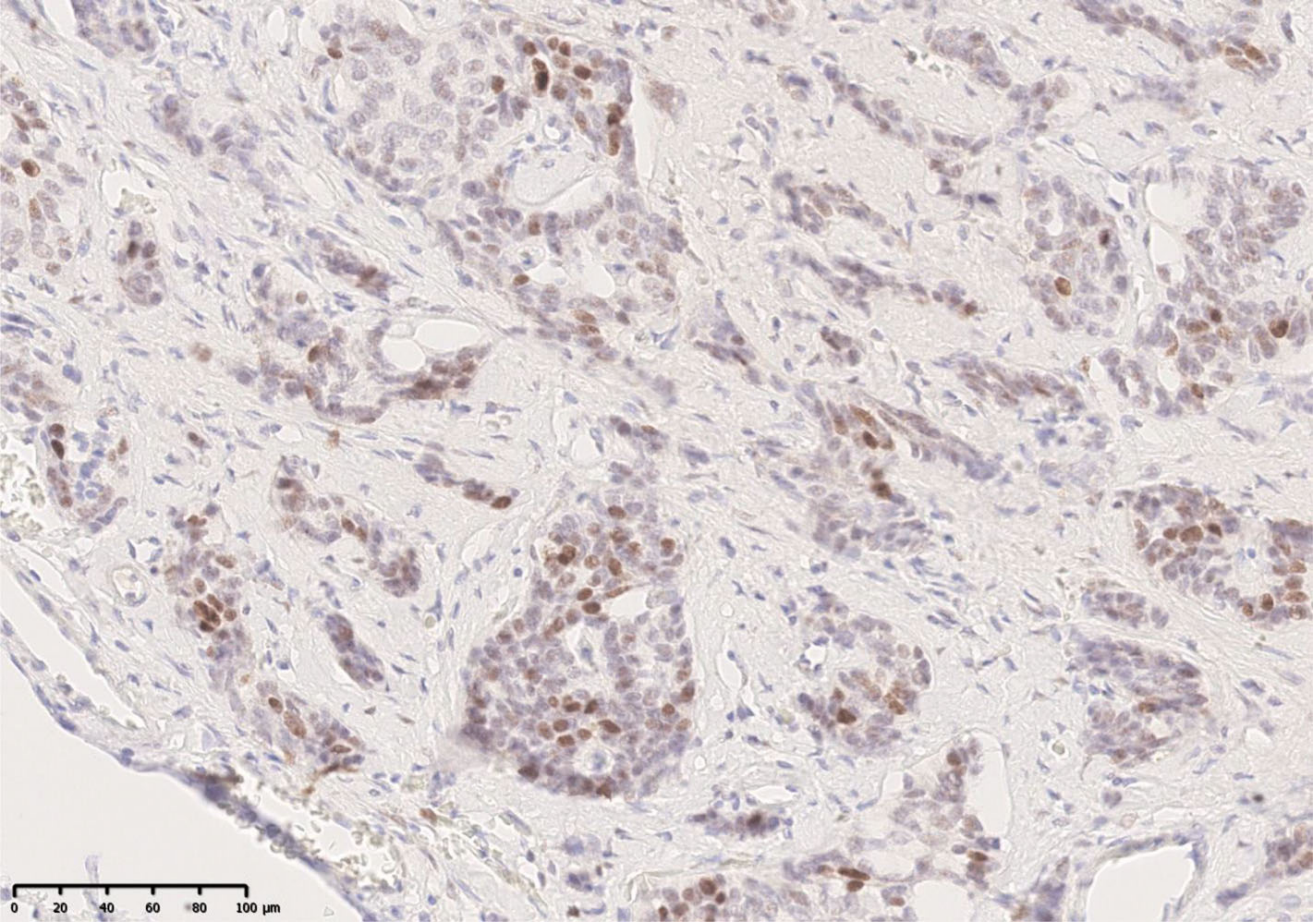
Description: Immunostaining for ER (IHC X20): 11%–33% of neoplastic nuclei show moderate positivity for ER and 50%–60% of neoplastic cells show weak positivity for ER (Quick Score* 5/8). *The Quick Score is claimed to be a reliable scoring system for evaluating ER expression in breast cancer. The Quick Score combines the percentage of positive cells and the intensity of the reaction product in most of the carcinoma. The 2 scores are added together for a final score with 8 possible values. Scores of 0 and 2 are considered negative. Scores of 3–8 are considered positive.

We also examined the oestrogen receptor (ER) and progesterone receptor (PR) status in 3 of the patients. Of note, patient nos. 4, 6 and 7 had tumours which stained positively for oestrogen receptors but not progesterone receptors (Figure 1).

## 4 DISCUSSION

In this cohort, progressive disease was reported in 35% (*n* = 7 out of 20 pregnancies) of the pregnancies in post‐partum period. Of these, 3 patients had a grade 1 midgut or pancreatic NET with a Ki 67 of <3%, 3 patients with 4 pregnancies had grade 2 pancreatic or renal NENs with Ki 67 of 5%–31%. While patients with residual NENs without active treatment could have further progression of the disease,^20^ two patients (one with a G2 pancreatic NEN on long‐acting somatostatin analogue, one with G1 pancreatic NET on subcutaneous octreotide) who were treated with somatostatin analogues also showed progressive disease. There is evidence to suggest that some NENs express oestrogen receptors^21^, ^22^ as well as progesterone receptors,^22^ and we were able to confirm this in 3 patients. Since pregnancy is characterised by high levels of oestrogen and progesterone secretion, whether activation of these receptors led to the tumour progression in the patients is an important hypothesis that needs further evaluation.

As described above, the gold standard for diagnosis of NENs is by histopathological examination. There are no data that indicate the histology differs between the pregnant and non‐pregnant women.^8^ Furthermore, prognosis based on the pathological staging of the tumours performed during pregnancy is not known to differ from the non‐pregnant women.^23^

In general, staging and surveillance of NENs in non‐pregnant women is carried out through cross‐sectional imaging modalities or through endoscopic examinations, where applicable (in gastric and rectal NENs).^11^ A number of patients with NENs diagnosed during pregnancy in this study (Table 1) were all diagnosed after detecting abnormal findings during routine obstetric ultrasound scanning (USS) performed during pregnancy. However, having suspected the presence of an NEN on USS, there remains the problem of utilising a more sensitive imaging modality which is safe during pregnancy. Guidelines on diagnostic imaging on pregnancy recommend serial USS and non‐contrast MRI as safe imaging modalities. Moreover, these guidelines recommend not to withhold computed tomography (CT) and nuclear imaging from pregnant women if these are deemed necessary in addition to USS and MRIs. However, the rare utilisation of contrast with MRI scans, CT scans and ^68^Gallium‐DOTATATE PET/CT scans should only be used in pregnant women where the putative benefits very clearly outweigh the risks.^24^, ^25^

In addition, patients should be also followed up by regular symptomatic assessment and physical examination, and the imaging should be used only if absolutely necessary. In the case of MEN1 syndrome patients with pituitary tumours, as in patient no. 9, they should be generally clinically assessed and undergo visual field assessments in each trimester in the case of pituitary macroadenomas. Non‐contrast MRI pituitary can be performed in the case of visual deterioration or worsening symptoms due to an enlarging adenoma.^23^, ^26^

Well‐differentiated NENs with SSTR‐positivity are usually treated with *SSTA* to enforce tumour stabilisation and ^177^Lu‐DOTATATE PRRT on progression.^27^ ^68^Ga‐DOTATATE PET/CT scans and octreotide scintigraphy mainly detect SSTR‐2 positive NENs.^11^ Apart from their anti‐proliferative and tumour‐stabilising uses, SSTA are also used to treat active syndromes, for example, carcinoid syndrome^28^ and symptoms in patients with functioning pancreatic NENs. If symptomatic relief is incomplete with the long acting SSTA (LA‐SSTA), additional short‐acting SSTA (SA‐SSTA), typically subcutaneous octreotide injections, can be used.^11^, ^29^ SSTA are also used in patients with GH‐secreting pituitary adenomas for symptom and tumour control,^30^, ^31^ such that experience with these agents during pregnancy can be extrapolated to patients with NENs. The action of the LA‐SSTA is exerted mainly through SSTR‐2 on the tumour cells.^27^ All types of SSTRs are expressed on the placental tissue and the umbilical cord although octreotide binding capacity at these sites is low.^32^ Octreotide is known to cross the placenta through passive diffusion^33^ but it is found in lower concentrations in the foetus and neonates in comparison to maternal blood.^32^ However, the elimination half‐life of octreotide in the neonates in considered to be significantly higher in comparison to adults.

The safety of both LA‐SSAs^27^, ^29^, ^30^, ^31^, ^33^ and SA‐SSAs^29^, ^32^ is evidenced by multiple case reports and case series indicating that there is no current evidence of teratogenicity or genotoxicity, although the number treated is small. Although most of these reports are based on their use in pregnant women with acromegaly,^33^ there is a case report claiming their safety in an ACTH‐secreting bronchial NEN.^34^ However, there is also evidence to suggest that somatostatin analogue injections might cause an acute reversible decrease in uterine artery blood flow.^32^ Moreover, a patient with acromegaly treated with octreotide LAR during pregnancy showed potential foetal growth restriction on the growth scan. The authors in this article also reported that with the reduction of the dose, normal growth was resumed until delivery.^30^ However, this case series also had 10 other patients with acromegaly who received LA‐SSAs during pregnancy without any foetal complications.^30^

In our study, 5 patients in 6 pregnancies were treated with LA‐SSTA (Octreotide LAR, Lanreotide autogel) and 4 of them continued treatment throughout pregnancy. Of these 6 pregnancies, 5 had successful deliveries at term with normal birth weight. Unfortunately, one patient had a preterm delivery with neonatal complications, related mostly due to prematurity. However, this neonate also had a congenital anomaly (ventriculomegaly) and had mild global developmental delay. Overall, in this cohort the rate of congenital anomalies was 5.5%, approximately doubled in comparison to the rate of congenital anomalies in the UK in 2018 (2.31%).^35^ However, the interpretation of this finding must be guarded due to the small sample size. One patient in our group who was diagnosed with an insulinoma was treated with SA‐SSTA (subcutaneous octreotide) daily injections during the first trimester and delivered a healthy full‐term baby, in keeping with the evidence from other studies.^29^, ^32^

Therefore, although some previous guidelines and publications in the management of acromegaly recommend stopping LA‐SSTA therapy before conception and advise starting these patients on short‐acting octreotide,^32^, ^36^ there is recent evidence to recommend the use of LA‐SSTA therapy in pregnant patients after carefully assessing the risks versus benefits with NENs in view of their actions as anti‐proliferative and anti‐secretory agents in NENs.^27^, ^29^, ^30^, ^31^, ^33^ There is no evidence base, but intuitively one might suggest that whether it was possible to hold SSTA during the first trimester this might be optimal and then reintroduce SSTA in the second trimester.

To the best of our knowledge, our study is the first study to compare TGRpc and TGRpp, and found that women who had stable or regressing NENs pre‐conceptually developed progressive disease and/or new lesions in the post‐pregnancy period. Approximately 30% of patients with a diagnosis of neuroendocrine tumours show immunohistochemical (IHC) expression of oestrogen (ER) and progesterone (PR) receptors. Previous studies have concluded that PR expression is primarily observed in pancreatic NEN and ER expression is more commonly observed in non‐pancreatic NEN.^37^ Therefore, it might be speculated that during pregnancy due to excessive oestrogen and progesterone exposure, there is a risk that these ER and/or PR‐expressing tumours could grow during pregnancy.

Finally, in our opinion the management of pregnant women should be subjected to a multidisciplinary team (MDT) and should also comprise maternal and foetal specialists other than the usual participants of NEN MDTs.^7^, ^8^ Selection of the most appropriate management strategy for these women should be based on the severity of symptoms, location of the tumour, grade and the stage of the tumour, gestational age and the availability of data on the safety of investigation and therapeutic modalities in pregnant women.^7^

## 5 CONCLUSIONS AND RECOMMENDATIONS

Although pregnancy with a concurrent diagnosis of NEN is rare, its incidence is increasing. These patients should be discussed in specialised NEN MDTs, also including an obstetrician and a neonatologist throughout their management during pregnancy. There are data suggesting the overall safety of SA‐SSTA and LA‐SSTA in pregnancy, and thus their use is indicated under close surveillance for their anti‐proliferative and anti‐secretory activities. Finally, further research is needed to assess the factors influencing progression of NENs during pregnancy, including the hormone responsiveness of NENs and the prevalence of oestrogen and progesterone receptor expression, because in our cohort there was a suggestion that pregnancy may enhance tumour growth in these patients.

## AUTHOR CONTRIBUTIONS

**Gowri M. Ratnayake:** Conceptualization; investigation; writing – original draft; methodology; writing – review and editing; formal analysis; project administration; resources; supervision; data curation. **Kalyan Mansukhbhai Shekhda:** Writing – review and editing; data curation; formal analysis. **Thomas Glover:** Data curation; formal analysis. **Yasser Al‐Obudi:** Data curation; formal analysis. **Aimee Hayes:** Writing – review and editing. **Panagiotis Armonis:** Writing – review and editing. **Dalvinder Mandair:** Writing – review and editing. **Bernard Khoo:** Writing – review and editing. **TuVinh Luong:** Data curation; writing – review and editing. **Christos Toumpanakis:** Writing – review and editing. **Ashley Grossman:** Writing – review and editing; supervision. **Martyn Caplin:** Conceptualization; writing – review and editing; supervision.

## CONFLICT OF INTEREST STATEMENT

The authors declare no conflicts of interest.

### PEER REVIEW

The peer review history for this article is available at https://www.webofscience.com/api/gateway/wos/peer‐review/10.1111/jne.13465.

## ETHICS STATEMENT

As this study was retrospective audit of practice, ethical approval was not required under the UK Policy Framework for Health and Social Care Practice.

## Data Availability

The data that support the findings of this study are available from the corresponding author upon reasonable request.
